# Trial Characteristics and Appropriateness of Statistical Methods Applied for Design and Analysis of Randomized School-Based Studies Addressing Weight-Related Issues: A Literature Review

**DOI:** 10.1155/2018/8767315

**Published:** 2018-06-25

**Authors:** Moonseong Heo, Singh R. Nair, Judith Wylie-Rosett, Myles S. Faith, Angelo Pietrobelli, Nancy R. Glassman, Sarah N. Martin, Stephanie Dickinson, David B. Allison

**Affiliations:** ^1^Department of Epidemiology and Population Health, Albert Einstein College of Medicine, Bronx, NY, USA; ^2^Department of Anesthesiology, Montefiore Medical Center, Bronx, NY, USA; ^3^Department of Counseling, School, and Educational Psychology, Graduate School of Education, University at Buffalo-SUNY, Buffalo, NY, USA; ^4^Department of Pediatrics, University of Verona, Verona, Italy; ^5^Pennington Biomedical Research Center, Baton Rouge, LA, USA; ^6^D. Samuel Gottesman Library, Albert Einstein College of Medicine, Bronx, NY, USA; ^7^Department of Epidemiology and Biostatistics, School of Public Health, Indiana University-Bloomington, Bloomington, IN, USA

## Abstract

**Objective:**

To evaluate whether clustering effects, often quantified by the intracluster correlation coefficient (ICC), were appropriately accounted for in design and analysis of school-based trials.

**Methods:**

We searched PubMed and extracted variables concerning study characteristics, power analysis, ICC use for power analysis, applied statistical models, and the report of the ICC estimated from the observed data.

**Results:**

*N*=263 papers were identified, and *N*=121 papers were included for evaluation. Overall, only a minority (21.5%) of studies incorporated ICC values for power analysis, fewer studies (8.3%) reported the estimated ICC, and 68.6% of studies applied appropriate multilevel models. A greater proportion of studies applied the appropriate models during the past five years (2013–2017) compared to the prior years (74.1% versus 63.5%, *p*=0.176). Significantly associated with application of appropriate models were a larger number of schools (*p*=0.030), a larger sample size (*p*=0.002), longer follow-up (*p*=0.014), and randomization at a cluster level (*p* < 0.001) and so were studies that incorporated the ICC into power analysis (*p*=0.016) and reported the estimated ICC (*p*=0.030).

**Conclusion:**

Although application of appropriate models has increased over the years, consideration of clustering effects in power analysis has been inadequate, as has report of estimated ICC. To increase rigor, future school-based trials should address these issues at both the design and analysis stages.

## 1. Introduction

Pediatric and adolescent obesity is a global concern as the range of its health consequence includes cardiovascular diseases, diabetes, poor quality of life, disability, mental health problems, and even adulthood mortality [1–5]. Despite this concern, the prevalence of pediatric and adolescent obesity has not decreased in the United States (US) or globally over the past several decades [6, 7]. In addition, financial and care burdens for preventing and treating pediatric obesity are substantial at both the individual or family level and societal or governmental level [8]. As such, countless trials have been conducted over the world to address pediatric and adolescent obesity and prevention, treatment, or diet guidelines based on evidence collected from the findings of those trials have been published [9, 10].

Many opine that schools represent “key settings” or “ideal settings” for obesity prevention or intervention, and numerous school-based studies have been conducted worldwide [11, 12]. The scope of interventions in school-based trials is broad ranging from education to health behaviors focusing on nutrition and physical activities [13–15]. However, the appropriate evaluation of intervention effectiveness critically hinges on proper trial design and statistical analysis of school-based trial data. Importantly, the data from school-based trials naturally form multiple levels of hierarchy. For example, when students are to be followed up multiple times for outcome measurements during the course of a study, repeatedly measured outcomes are nested within students who are in turn nested within schools. This hierarchical nature forms a three-level data structure so that clustering effects, also known as design effects, of outcome data at the school and student levels should be taken into account not only at the design stage but also at the analysis stage.

The critical issue is that required sample sizes are likely to be underestimated if such clustering effects are not taken into account [16–20]. This is especially so when the interventions are assigned at the highest level of data hierarchy. Likewise, the standard errors of estimated intervention effects at the analytic stage are smaller than what it should be and so are *p* values, likely increasing the type I error rate [21]. Anecdotally, errors in neglecting such clustering seem not uncommon [22].

The primary aim of this review is to evaluate statistical methods applied to published school-based randomized trials addressing weight issues. The evaluation is focused on assessing appropriateness of power analysis and applied statistical methods as to whether clustering effects often represented by correlations of subject outcomes within schools, which is also known as the intracluster (or intraclass) correlation coefficient (ICC) [23], are properly accounted for in the design and analysis of school-based trials. Specifically, we aim to examine (1) whether study characteristics are different between the past five years (2013–2017) and the prior years (1995–2012), that is, between pre- and postera of the updated, extended CONSORT statement for cluster randomized trials published in the year 2012 [24] and (2) whether appropriateness of statistical methods is associated with study characteristics.

## 2. Methods

### 2.1. Search Strategy

We searched PubMed for papers meeting the following inclusion criteria:

- Published in journals indexed in PubMed
- From the PubMed inception year
- Randomized school-based trials
- Written in English
- Outcomes involving weight-related issues
- Subjects of age younger than 25 years that include young adults.

To this end, we applied a Boolean algebraic combination of MeSH (*Me*dical *S*ubject *H*eadings) as follows: (“Body Weight”[MeSH] OR “Body Mass Index”[MeSH]) AND “Schools”[MeSH] AND (Randomized Controlled Trial[ptyp] AND English[lang] AND (“adolescent”[MeSH Terms] OR “child”[MeSH Terms:noexp] OR “child, preschool”[MeSH Terms] OR “young adult”[MeSH Terms])). With this search strategy, we identified a total of 263 papers as of December 8, 2017, and the earliest publication year was 1995.

### 2.2. Exclusion Criteria

We retrieved full texts of all of those papers, and two authors (MH and SN) applied the following exclusion criteria for the review:

- Protocol paper.
- Not a randomized study.
- Not a school-based intervention.
- Analysis of subjects of age beyond the range.
- Outcome is not related to weight issues.
- Baseline or subgroup analysis of a parent study.
- Single-school trials.

When the two raters' ratings were not concordant due to unclear descriptions in the text regarding study design parameters or analytic methods, the rating was resolved by consensus between them.

### 2.3. Extracted Variables and Classifications

We extracted the following study characteristic variables from the included studies: the publication year (past 5 years (2013–2017) versus the prior years (1995–2012)); number of schools; analytic sample sizes of individuals, which are the size of the completers at the final follow-up when available; randomization level (cluster versus individual); length of follow-up in months (<12 versus ≥12 months); number of repeated measurements including baseline (2 versus >2); location of the trial (the US versus others); and types of schools (preschool, elementary/primary, middle, and high school, and college).

As per the randomization level, the “cluster” classification category includes any higher level units in which all subjects received the identical interventions or treatments. Therefore, the cluster unit could be schools, classes, health centers, communities, camps, and so on. Nevertheless, we extracted the number of involved schools, instead of the number of the various randomized cluster units, for the number of schools variable. If randomization occurred at the individual/student level within schools or other cluster units, we classified the randomization level for such trials as “individual.” When multiple types of schools are involved in a trial, we classified such cases into the school type of the lowest level. For instance, if a trial involved preschool and primary schools, then this case was classified as “preschool” for the school type.

With respect to the presence of power analysis and appropriateness of applied statistical methods, we extracted the report of power analysis (yes versus no); consideration of the intracluster correlation coefficient (ICC) for power analysis (yes versus no); primary statistical analysis models (multilevel versus no); and the report of the ICC estimated from statistical analysis (yes versus no). If the description of power analysis or sample size determination referred to a protocol paper and was not provided in the text to an evaluable extent, we classified such cases as no report of power analysis. As the terminologies of statistical models are not standardized across the papers, we classified the following models as “multilevel” models in which a “clustering” effect at some level of the data hierarchy had presumably been taken into account in the analysis: generalized mixed-effects model, mixed-effects model, linear mixed-effects model, multilevel mixed-effects model, hierarchical models, random-intercept models, mixed models, logistic mixed-effects models, generalized estimating equation models, and the likes. We consider the application of those multilevel analyses appropriate. Other models that do not take the clustering effects into account were classified as “no” multilevel model: for example, paired *t*-test, chi-square test, analysis of variance (ANOVA), analysis of covariance (ANCOVA), linear or logistic regression models, and repeated-measures AN(C)OVA at the individual level.

### 2.4. Statistical Analysis

Descriptive statistics are provided in terms of frequency, percentages, median, and the first (Q1) and the third (Q3) quartiles. There were three missing values for the number of schools, and studies with those missing values were excluded from analyses involving the number of schools. The follow-up length and number of measurements are analyzed as both continuous and dichotomized variables. Comparisons of study characteristics, the presence of power analysis, and appropriateness of statistical methods between the publication years 2013–2017 versus 1995–2012 are made using Wilcoxon rank-sum tests or Fisher exact tests. The same sets of these tests are also applied to testing associations of study characteristics and the presence of power analysis with application of appropriate multilevel analysis. Statistical significance is declared at *p* < 0.05 (two-tailed). SAS v9.4 was used for all analyses.

## 3. Results

### 3.1. Reasons for Exclusion

The application of the exclusion criteria resulted in 121 papers (Supplementary Materials (available here)) with 1995 as the earliest publication year after exclusion of 142 papers. The reasons for exclusions are displayed in Table 1. Although it is possible that a study was excluded for multiple reasons, the most common reason was due to single-school trials (34.5%) followed by protocol papers (25.4%).

### 3.2. Study Characteristics between the Past Five Years and the Prior Years

Detailed results are presented in Table 2. There were 63 (52%) and 58 (48%) studies included for analysis between the years 2013–2017 versus 1995–2012, respectively. None of the listed study characteristics, except the school type, significantly differ between those time frames. We note that the initial extended CONSORT guideline statement for cluster randomized trials was published in the year 2004 [25], but we did not further categorize years into three time frames including those before 2005 due to a relatively small number of included studies during that time period (*N*=19 or 7.3%). Overall, the vast majority of studies randomized interventions at a cluster level (89.3%), and the analytic sample size is relatively large with a median of 620 students. The majority (58.9%) of studies had only two measurements: one at baseline or before intervention and the other at the end of the trial/follow-up or after intervention. Most (57.0%) of the studies were conducted outside the US, and more so for the past five years compared to the prior years (63.8% versus 50.8%, *p*=0.198). The number of countries outside the US was 30 from all continents; one study involved six European countries. The primary/elementary schools most often served as experimental settings (68.6%); however, those schools were significantly less utilized during the past 5 years compared to the prior years (56.9% versus 79.4%, *p*=0.010). Perhaps for this reason, the distributions of overall types of schools are significantly different between years (*p*=0.038).

### 3.3. Power Analysis, ICC, and Multilevel Models between the Past Five Years and the Prior Years

Results from the comparisons of power analysis and application of multilevel models between years are presented in Table 3. A minority (43.0%) of the studies reported power analysis adequately in the text, and the proportion was not significantly different between the past five years and the prior years (48.3% versus 38.1%, *p*=0.258). However, most studies applied multilevel analytic methods (68.6%), and the proportion was greater for the past five years (74.1% versus 63.5%, *p*=0.176). About one-fifth of studies (21.5%) took the ICC into consideration for power analysis, and the past five years did not see a significant increase in this proportion. A smaller number of studies (8.3%) reported ICCs estimated from the statistical analysis of trial data, and the proportions decreased, though not significantly, during the past five years.

### 3.4. Association between Multilevel Analysis and Study Characteristics

As presented in Table 4, a majority of study characteristics are associated with the application of appropriate multilevel statistical models. Specifically, a larger number of schools (*p*=0.030), a larger size of the analytic sample (*p*=0.002), longer follow-up (*p*=0.014), and randomization at a cluster level (*p* < 0.001) were all significantly associated with the application of appropriate models. The location of trials was not significantly associated with the appropriateness (*p*=1.000). The studies with appropriate multilevel models were more likely to have incorporated the ICC into power analysis (*p*=0.016) and reported the ICC from the analysis of trial data (*p*=0.030). As should be the case, no studies with non-multilevel models reported ICC estimates. However, even among studies with multilevel models, only a minority (12.1%) reported ICC estimates.

## 4. Discussion

The primary finding of this review is that the proportion of studies which failed to apply multilevel models to analyzing school-based data appears to be relatively high at 31.4%. In addition, even if multilevel models were applied, specification of levels of clustering effects was rarely described (data not shown). For instance, clustering effects of only the highest-level units of data hierarchy seem to have been taken into account, ignoring additional potential clustering effects of lower-levels units. Taken together, significance of findings based on *p* values from those studies might have possibly been falsely declared especially when the *p* values are close to 0.05. Furthermore, less than half of all studies reported power analysis, and approximately one-fifth of all studies, or equivalently about half of the studies that reported power analysis, took the ICC into consideration for power analysis. Therefore, it is likely that a majority of studies might have been underpowered implying that even if the study findings are significant, one cannot rule out the possibility of type I error.

The magnitudes of ICC for power analysis were mostly low, and rationales for such a hypothesized ICC are seldom clearly described. The dearth of rationale might have been due to the lack of information regarding ICC estimates, published or not, pertinent to their studies. This is reflected on the very low proportion of studies (8.3%) that reported ICCs estimated from their data analysis. To this end, the reporting of the ICC estimated from data analysis would be critical for designing future studies with adequate sample sizes. Even if the ICC appears to be very small, it needs to be accounted for power analysis. For example, when the number of students is as small as 30 for each school, the number of required schools with ICC = 0.01 increases by 29% for the same power, compared to when ICC = 0, regardless of hypothesized effect sizes. Therefore, the impact of a small ICC on the sample size could be substantial.

Detailed design characteristics are often referred to protocol papers published earlier, and adequacy of power analysis was not evaluable in the text of outcome papers. Classification of such outcome papers as no report of power analysis might have underestimated the proportion of studies with power analysis because the protocol papers might have properly reported power analysis. However, we surmise that it is fairly rare for a school-based study to be conducted exactly as planned or designed in the protocol papers as school environments are dynamic and changes may alter aspects of the design or analysis plans including sample size determinations/power analysis, statistical methods, and outcome parameters. If outcomes papers do not clearly delineate these aspects, it may be unclear to know whether the power analysis in the earlier protocol paper would be appropriate for the applied statistical methods for the analysis of trial outcomes. We believe that this issue would be a research topic worthy to investigate. After all, the following key design elements should be reported in the outcome analysis papers: target power, significance level, hypothesized effect sizes and ICCs and their rationales, the number of clusters, the number of levels, anticipated attrition rates, and the planned sample size of the subjects. This description can enable readers to effectively and clearly evaluate whether the study has been analyzed as designed, not being forced to compare with detailed elements described in the protocol papers.

Many trials are excluded from evaluation due to the utilization of single schools. These studies are mostly college-based trials likely because multicollege trials may be difficult to conduct compared to the other types of schools. Nonetheless, it is not possible to take the clustering effect into account not only for power analysis but also for statistical analysis, nor is it possible to estimate ICCs from data analysis from single-school/cluster studies. Although it could bring up an issue as to what extent clustering effects should be considered in general, this limitation should be addressed in regard to limited generalizability or transportability of the findings from single-school trials. Identical or similar findings from replicate studies in other school settings would validate the findings.

Our findings also have implications for doctoral programs training future obesity researchers, particularly those conducting school-based interventions. The programs include but are not limited to health, clinical, and school psychology subspecialties. Researchers should ensure that the most rigorous and appropriate methodologies, including issues related to clustering addressed in this study, are included as part of the core curriculum. In this way, students learn early in their careers the practice of reporting such information. To this point, the American Psychological Association (APA) recently released two APA Publications and Communications Board Task Force reports addressing standards for reporting study results. Separate reports were made for quantitative studies [26], as well as qualitative, meta-analytic, and mixed-methods research [27].

Although our review is confined to school-based trials, the findings may be applicable to other cluster randomized trials using different settings and different types of interventions and treatments in other research areas [28–31]. Collectively, therefore, it would be ideal for reports of cluster randomized trials in general to adhere to the aforementioned 2012 updated CONSORT guideline [24] for both outcome and protocol papers. This guideline proposes all the design and analysis elements that should be reported in a standardized manner on manuscripts based on cluster randomized trials. If reports are standardized, it will be beneficial for researchers not only to plan or design school-based trials or other cluster randomized trials but also to more efficiently conduct systematic reviews and meta-analyses with greater statistical power and clearer transparencies.

### 4.1. Limitations

There are limitations that should be counted when interpreting results from this review. First, the search strategy was rather incomprehensive including only PubMed papers, and the keywords for search may be coarse. Therefore, studies that would have been eligible and added to our evaluations might not have been captured, and the scopes of potentially missing studies are unknown. As is the case for review papers in general, subjective ratings may not be completely avoided even if efforts are placed in minimizing misclassification errors. For instance, we did not evaluate whether potential confounding factors were appropriately controlled for in the data analysis. This evaluation might rely more on subjective judgments with substantial knowledge on the research topics under study and also might be difficult to reach a consensus. Lastly, again, the proportion of the studies with the reported power analysis might have been underestimated because studies that referred detailed power analysis to a protocol paper without a minimum level of description in the text were counted as papers with no report of power analysis.

## 5. Conclusions

In conclusion, the extent of the application of multilevel models to analyzing school-based trials appears to have so far been inadequate. Key elements such as the hypothesized ICC for power analysis or sample size determinations and the reported ICC estimated from data analyses of school-based trials are missing for a majority of studies. Future school-based trials should specifically address these issues at both design and analysis stages, preferably adhering to the extended CONSORT guideline to increase rigor and reproducibility of experimental settings and study findings. Clinical implications drawn based on the outcomes from school-based trials with rigorously well-performed design, conduct, and analysis would be the most useful to advance knowledge for preventing and treating pediatric and adolescent obesity epidemic.

## Figures and Tables

**Table 1:** Reasons for exclusion.

Reasons	*N*	%
Single-school trial	49	34.5
Protocol paper	36	25.4
Baseline or subgroup analysis of a parent study	27	19.0
Not a randomized trial	18	12.7
Students of age beyond the age range	7	4.9
Not a school-based trial	5	3.5
Total	142	

*Note*. Although it is possible that studies could be excluded for multiple reasons, we classified the reasons in a mutually exclusive manner.

**Table 2:** Study characteristics by study years: median (Q1, Q3), *N* (%).

Study characteristics	All years (*N*=121)	1995–2012 (*N*=63)	2013–2017 (*N*=58)	*p* value
Number of schools^*∗*^	13 (7, 28)	14 (8, 31)	12 (6, 20)	0.434
Analytic sample size	620 (340, 1182)	670 (407, 1295)	610 (310, 1083)	0.269
Follow-up length in months	12 (6, 24)	12 (6, 24)	12 (5, 20)	0.078
Number of repeated measurements	2 (2, 3)	2 (2, 3)	2 (2, 3)	0.098
Randomization at a cluster level	108 (89.3%)	57 (90.5%)	51 (87.9%)	0.772
Follow-up ≥1 year	68 (56.2%)	38 (60.3%)	30 (51.7%)	0.365
Two measurements including baseline	70 (57.9%)	32 (50.8%)	38 (65.5%)	0.140
Trials conducted outside the US	69 (57.0%)	32 (50.8%)	37 (63.8%)	0.198
School type				0.038
Preschool	11 (9.1%)	3 (4.8%)	8 (13.8%)	
Primary/elementary school	83 (68.6%)	50 (79.4%)	33 (56.9%)	
Middle school	20 (16.5%)	7 (11.1%)	13 (22.4%)	
High school	5 (4.1%)	3 (4.8%)	2 (3.5%)	
College	2 (1.7%)	0 (0.0%)	2 (3.5%)	

^*∗*^ *N*=3 is missing.

**Table 3:** Power analysis and statistical methods by study years: *N* (%).

Power analysis statistical methods	All years (*N*=121)	1995–2012 (*N*=63)	2013–2017 (*N*=58)	*p* value
Power analysis conducted and reported	52 (43.0%)	24 (38.1%)	28 (48.3%)	0.258
ICC taken into account for power analysis	26 (21.5%)	13 (20.6%)	13 (22.4%)	0.828
Multilevel analysis performed	83 (68.6%)	40 (63.5%)	43 (74.1%)	0.176
ICC estimated from the analysis reported	10 (8.3%)	7 (11.1%)	3 (5.2%)	0.327

**Table 4:** Association between multilevel analysis and study characteristics and power analysis: median (Q1, Q3), *N* (%).

Study characteristics	Multilevel analysis		*p* value
	Yes (*N*=83)	No (*N*=38)	
Number of schools^*∗*^	16 (8, 32)	10 (6, 18)	0.030
Analytic sample size	816 (432, 1323)	472 (181, 869)	0.002
Follow-up length in months	12 (6, 24)	7 (4, 18)	0.014
Number of measurements	2 (2, 3)	2 (2, 3)	0.558
Randomization at a cluster level	82 (98.8%)	26 (68.4%)	<0.001
Follow-up ≥1 year	52 (62.7%)	16 (42.1%)	0.048
Two measurements including baseline	47 (56.6%)	23 (60.5%)	0.843
Trials conducted outside the US	47 (56.6%)	22 (57.9%)	1.000

*Power analysis and ICC*			
Power analysis conducted and reported	39 (47.0%)	13 (34.2%)	0.236
ICC taken into account for power analysis	23 (27.7%)	3 (7.9%)	0.016
ICC estimated from the analysis reported	10 (12.1%)	0 (0.0%)	0.030

^*∗*^ *N*=3 is missing.

## Data Availability

The data analyzed for the present analysis along with programming codes will be available upon reasonable request.

## Supplementary Materials

Supplementary Materials This material contains the list of 121 papers that we reviewed and evaluated for this paper.
