# Supplementary material for: Trial Characteristics and Appropriateness of Statistical Methods Applied for Design and Analysis of Randomized School-Based Studies Addressing Weight-Related Issues: A Literature Review
Source: J Obes. 2018 Jun 25;2018:8767315. doi: 10.1155/2018/8767315 (PMC6036807; doi:10.1155/2018/8767315)
Supplement: Supplementary Materials — This material contains the list of 121 papers that we reviewed and evaluated for this paper. [file 8767315.f1.docx]

**Supplementary Material: List of 121 papers included for evaluations**

1. Abood DA, Black DR, Coster DC. Evaluation of a school-based teen obesity prevention minimal intervention. Journal of nutrition education and behavior. 2008;40(3):168-74.

2. Alaimo K, Oleksyk SC, Drzal NB, Golzynski DL, Lucarelli JF, Wen Y, et al. Effects of changes in lunch-time competitive foods, nutrition practices, and nutrition policies on low-income middle-school children's diets. Childhood obesity (Print). 2013;9(6):509-23.

3. Amini M, Djazayery A, Majdzadeh R, Taghdisi MH, Sadrzadeh-Yeganeh H, Abdollahi Z, et al. A School-Based Intervention to Reduce Excess Weight in Overweight and Obese Primary School Students. Biological research for nursing. 2016;18(5):531-40.

4. Andersen R, Biltoft-Jensen A, Christensen T, Andersen EW, Ege M, Thorsen AV, et al. Dietary effects of introducing school meals based on the New Nordic Diet - a randomised controlled trial in Danish children. The OPUS School Meal Study. Br J Nutr. 2014;111(11):1967-76.

5. Andrade S, Lachat C, Ochoa-Aviles A, Verstraeten R, Huybregts L, Roberfroid D, et al. A school-based intervention improves physical fitness in Ecuadorian adolescents: a cluster-randomized controlled trial. The international journal of behavioral nutrition and physical activity. 2014;11:153.

6. Annesi JJ, Smith AE, Tennant GA. Effects of a cognitive-behaviorally based physical activity treatment for 4- and 5-year-old children attending US preschools. International journal of behavioral medicine. 2013;20(4):562-6.

7. Annesi JJ, Vaughn LL. Evidence-Based Referral: Effects of the Revised "Youth Fit 4 Life" Protocol on Physical Activity Outputs. The Permanente journal. 2015;19(3):48-53.

8. Ash DM, Tatala SR, Frongillo EA, Jr., Ndossi GD, Latham MC. Randomized efficacy trial of a micronutrient-fortified beverage in primary school children in Tanzania. Am J Clin Nutr. 2003;77(4):891-8.

9. Ask AS, Hernes S, Aarek I, Vik F, Brodahl C, Haugen M. Serving of free school lunch to secondary-school pupils - a pilot study with health implications. Public Health Nutr. 2010;13(2):238-44.

10. Atkinson MJ, Wade TD. Mindfulness-based prevention for eating disorders: A school-based cluster randomized controlled study. Int J Eat Disord. 2015;48(7):1024-37.

11. Bacardi-Gascon M, Perez-Morales ME, Jimenez-Cruz A. A six month randomized school intervention and an 18-month follow-up intervention to prevent childhood obesity in Mexican elementary schools. Nutr Hosp. 2012;27(3):755-62.

12. Barbosa Filho VC, da Silva KS, Mota J, Vieira NF, Gubert FD, Lopes AD. "For whom was it effective?" Moderators of the effect of a school-based intervention on potential physical activity determinants among Brazilian students. Prev Med. 2017;97:80-5.

13. Beets MW, Turner-McGrievy B, Weaver RG, Huberty J, Moore JB, Ward DS, et al. Intervention leads to improvements in the nutrient profile of snacks served in afterschool programs: a group randomized controlled trial. Translational behavioral medicine. 2016;6(3):329-38.

14. Bjelland M, Bergh IH, Grydeland M, Klepp KI, Andersen LF, Anderssen SA, et al. Changes in adolescents' intake of sugar-sweetened beverages and sedentary behaviour: results at 8 month mid-way assessment of the HEIA study--a comprehensive, multi-component school-based randomized trial. The international journal of behavioral nutrition and physical activity. 2011;8:63.

15. Brandstetter S, Klenk J, Berg S, Galm C, Fritz M, Peter R, et al. Overweight prevention implemented by primary school teachers: a randomised controlled trial. Obes Facts. 2012;5(1):1-11.

16. Caballero B, Clay T, Davis SM, Ethelbah B, Rock BH, Lohman T, et al. Pathways: a school-based, randomized controlled trial for the prevention of obesity in American Indian schoolchildren. Am J Clin Nutr. 2003;78(5):1030-8.

17. Christiansen LB, Toftager M, Boyle E, Kristensen PL, Troelsen J. Effect of a school environment intervention on adolescent adiposity and physical fitness. Scand J Med Sci Sports. 2013;23(6):e381-9.

18. Cohen JF, Kraak VI, Choumenkovitch SF, Hyatt RR, Economos CD. The CHANGE study: a healthy-lifestyles intervention to improve rural children's diet quality. J Acad Nutr Diet. 2014;114(1):48-53.

19. Coleman KJ, Shordon M, Caparosa SL, Pomichowski ME, Dzewaltowski DA. The healthy options for nutrition environments in schools (Healthy ONES) group randomized trial: using implementation models to change nutrition policy and environments in low income schools. The international journal of behavioral nutrition and physical activity. 2012;9:80.

20. Collard DC, Verhagen EA, Chinapaw MJ, Knol DL, van Mechelen W. Effectiveness of a school-based physical activity injury prevention program: a cluster randomized controlled trial. Arch Pediatr Adolesc Med. 2010;164(2):145-50.

21. Crepinsek MK, Singh A, Bernstein LS, McLaughlin JE. Dietary effects of universal-free school breakfast: findings from the evaluation of the school breakfast program pilot project. J Am Diet Assoc. 2006;106(11):1796-803.

22. Crespo NC, Elder JP, Ayala GX, Slymen DJ, Campbell NR, Sallis JF, et al. Results of a multi-level intervention to prevent and control childhood obesity among Latino children: the Aventuras Para Ninos Study. _nn Behav Med. 2012;43(1):84-100.

23. Cunha DB, de Souza Bda S, Pereira RA, Sichieri R. Effectiveness of a randomized school-based intervention involving families and teachers to prevent excessive weight gain among adolescents in Brazil. PLoS One. 2013;8(2):e57498.

24. Davis SM, Clay T, Smyth M, Gittelsohn J, Arviso V, Flint-Wagner H, et al. Pathways curriculum and family interventions to promote healthful eating and physical activity in American Indian schoolchildren. Prev Med. 2003;37(6 Pt 2):S24-34.

25. Davis SM, Myers OB, Cruz TH, Morshed AB, Canaca GF, Keane PC, et al. CHILE: Outcomes of a group randomized controlled trial of an intervention to prevent obesity in preschool Hispanic and American Indian children. Prev Med. 2016;89:162-8.

26. De Bock F, Breitenstein L, Fischer JE. Positive impact of a pre-school-based nutritional intervention on children's fruit and vegetable intake: results of a cluster-randomized trial. Public Health Nutr. 2012;15(3):466-75.

27. DeVault N, Kennedy T, Hermann J, Mwavita M, Rask P, Jaworsky A. It's all about kids: preventing overweight in elementary school children in Tulsa, OK. J Am Diet Assoc. 2009;109(4):680-7.

28. Dewar DL, Morgan PJ, Plotnikoff RC, Okely AD, Collins CE, Batterham M, et al. The nutrition and enjoyable activity for teen girls study: a cluster randomized controlled trial. Am J Prev Med. 2013;45(3):313-7.

29. Donnelly JE, Greene JL, Gibson CA, Smith BK, Washburn RA, Sullivan DK, et al. Physical Activity Across the Curriculum (PAAC): a randomized controlled trial to promote physical activity and diminish overweight and obesity in elementary school children. Prev Med. 2009;49(4):336-41.

30. Dunton G, Ebin VJ, Efrat MW, Efrat R, Lane CJ, Plunkett S. The Use of Refundable Tax Credits to Increase Low-Income Children's After-School Physical Activity Level. Journal of physical activity & health. 2015;12(6):840-53.

31. Efrat MW. Exploring effective strategies for increasing the amount of moderate-to-vigorous physical activity children accumulate during recess: a quasi-experimental intervention study. J Sch Health. 2013;83(4):265-72.

32. Evans A, Ranjit N, Hoelscher D, Jovanovic C, Lopez M, McIntosh A, et al. Impact of school-based vegetable garden and physical activity coordinated health interventions on weight status and weight-related behaviors of ethnically diverse, low-income students: Study design and baseline data of the Texas, Grow! Eat! Go! (TGEG) cluster-randomized controlled trial. BMC Public Health. 2016;16:973.

33. Ezendam NP, Brug J, Borsboom G, van Empelen P, Oenema A. Differential effects of the computer-tailored FATaintPHAT programme on dietary behaviours according to sociodemographic, cognitive and home environmental factors. Public Health Nutr. 2014;17(2):431-9.

34. Ezendam NP, Brug J, Oenema A. Evaluation of the Web-based computer-tailored FATaintPHAT intervention to promote energy balance among adolescents: results from a school cluster randomized trial. Arch Pediatr Adolesc Med. 2012;166(3):248-55.

35. Fitzgibbon ML, Stolley MR, Schiffer L, Kong A, Braunschweig CL, Gomez-Perez SL, et al. Family-based hip-hop to health: outcome results. Obesity (Silver Spring). 2013;21(2):274-83.

36. Fitzgibbon ML, Stolley MR, Schiffer LA, Braunschweig CL, Gomez SL, Van Horn L, et al. Hip-Hop to Health Jr. Obesity Prevention Effectiveness Trial: postintervention results. Obesity (Silver Spring). 2011;19(5):994-1003.

37. Francis M, Nichols SS, Dalrymple N. The effects of a school-based intervention programme on dietary intakes and physical activity among primary-school children in Trinidad and Tobago. Public Health Nutr. 2010;13(5):738-47.

38. Gatto NM, Martinez LC, Spruijt-Metz D, Davis JN. LA sprouts randomized controlled nutrition, cooking and gardening programme reduces obesity and metabolic risk in Hispanic/Latino youth. Pediatric obesity. 2017;12(1):28-37.

39. Gentile DA, Welk G, Eisenmann JC, Reimer RA, Walsh DA, Russell DW, et al. Evaluation of a multiple ecological level child obesity prevention program: Switch what you Do, View, and Chew. BMC medicine. 2009;7:49.

40. Giannaki CD, Aphamis G, Tsouloupas CN, Ioannou Y, Hadjicharalambous M. An eight week school-based intervention with circuit training improves physical fitness and reduces body fat in male adolescents. J Sports Med Phys Fitness. 2016;56(7-8):894-900.

41. Gortmaker SL, Peterson K, Wiecha J, Sobol AM, Dixit S, Fox MK, et al. Reducing obesity via a school-based interdisciplinary intervention among youth: Planet Health. Arch Pediatr Adolesc Med. 1999;153(4):409-18.

42. Greening L, Harrell KT, Low AK, Fielder CE. Efficacy of a school-based childhood obesity intervention program in a rural southern community: TEAM Mississippi Project. Obesity (Silver Spring). 2011;19(6):1213-9.

43. Grillich L, Kien C, Takuya Y, Weber M, Gartlehner G. Effectiveness evaluation of a health promotion programme in primary schools: a cluster randomised controlled trial. BMC Public Health. 2016;16:679.

44. Grydeland M, Bergh IH, Bjelland M, Lien N, Andersen LF, Ommundsen Y, et al. Intervention effects on physical activity: the HEIA study - a cluster randomized controlled trial. The international journal of behavioral nutrition and physical activity. 2013;10:17.

45. Gutin B, Yin Z, Johnson M, Barbeau P. Preliminary findings of the effect of a 3-year after-school physical activity intervention on fitness and body fat: the Medical College of Georgia Fitkid Project. International journal of pediatric obesity : IJPO : an official journal of the International Association for the Study of Obesity. 2008;3 Suppl 1:3-9.

46. Habib-Mourad C, Ghandour LA, Moore HJ, Nabhani-Zeidan M, Adetayo K, Hwalla N, et al. Promoting healthy eating and physical activity among school children: findings from Health-E-PALS, the first pilot intervention from Lebanon. BMC Public Health. 2014;14:940.

47. Haerens L, Deforche B, Maes L, Stevens V, Cardon G, De Bourdeaudhuij I. Body mass effects of a physical activity and healthy food intervention in middle schools. Obesity (Silver Spring). 2006;14(5):847-54.

48. Harrington KF, Kohler CL, McClure LA, Franklin FA. Fourth graders' reports of fruit and vegetable intake at school lunch: does treatment assignment affect accuracy? J Am Diet Assoc. 2009;109(1):36-44.

49. Hill LJ, Williams JH, Aucott L, Thomson J, Mon-Williams M. How does exercise benefit performance on cognitive tests in primary-school pupils? Dev Med Child Neurol. 2011;53(7):630-5.

50. Iannotti L, Dulience SJ, Joseph S, Cooley C, Tufte T, Cox K, et al. Fortified Snack Reduced Anemia in Rural School-Aged Children of Haiti: A Cluster-Randomized, Controlled Trial. PLoS One. 2016;11(12):e0168121.

51. Jago R, Sebire SJ, Davies B, Wood L, Edwards MJ, Banfield K, et al. Randomised feasibility trial of a teaching assistant led extracurricular physical activity intervention for 9 to 11 year olds: Action 3:30. The international journal of behavioral nutrition and physical activity. 2014;11:114.

52. Jansen W, Borsboom G, Meima A, Zwanenburg EJ, Mackenbach JP, Raat H, et al. Effectiveness of a primary school-based intervention to reduce overweight. International journal of pediatric obesity : IJPO : an official journal of the International Association for the Study of Obesity. 2011;6(2-2):e70-7.

53. Kafatos A, Manios Y, Moschandreas J. Health and nutrition education in primary schools of Crete: follow-up changes in body mass index and overweight status. Eur J Clin Nutr. 2005;59(9):1090-2.

54. Kain J, Concha F, Moreno L, Leyton B. School-based obesity prevention intervention in Chilean children: effective in controlling, but not reducing obesity. Journal of obesity. 2014;2014:618293.

55. Kattelmann KK, Bredbenner CB, White AA, Greene GW, Hoerr SL, Kidd T, et al. The effects of Young Adults Eating and Active for Health (YEAH): a theory-based Web-delivered intervention. Journal of nutrition education and behavior. 2014;46(6):S27-41.

56. Katz DL, Katz CS, Treu JA, Reynolds J, Njike V, Walker J, et al. Teaching healthful food choices to elementary school students and their parents: the Nutrition Detectives program. J Sch Health. 2011;81(1):21-8.

57. Kelly EB, Parra-Medina D, Pfeiffer KA, Dowda M, Conway TL, Webber LS, et al. Correlates of physical activity in black, Hispanic, and white middle school girls. Journal of physical activity & health. 2010;7(2):184-93.

58. Kesztyus D, Lauer R, Kesztyus T, Kilian R, Steinacker JM. Costs and effects of a state-wide health promotion program in primary schools in Germany - the Baden-Wurttemberg Study: A cluster-randomized, controlled trial. PLoS One. 2017;12(2):e0172332.

59. Kipping RR, Howe LD, Jago R, Campbell R, Wells S, Chittleborough CR, et al. Effect of intervention aimed at increasing physical activity, reducing sedentary behaviour, and increasing fruit and vegetable consumption in children: active for Life Year 5 (AFLY5) school based cluster randomised controlled trial. BMJ. 2014;348:g3256.

60. Kobel S, Wirt T, Schreiber A, Kesztyus D, Kettner S, Erkelenz N, et al. Intervention effects of a school-based health promotion programme on obesity related behavioural outcomes. Journal of obesity. 2014;2014:476230.

61. Kocken PL, Eeuwijk J, Van Kesteren NM, Dusseldorp E, Buijs G, Bassa-Dafesh Z, et al. Promoting the purchase of low-calorie foods from school vending machines: a cluster-randomized controlled study. J Sch Health. 2012;82(3):115-22.

62. Lent MR, Vander Veur SS, McCoy TA, Wojtanowski AC, Sandoval B, Sherman S, et al. A randomized controlled study of a healthy corner store initiative on the purchases of urban, low-income youth. Obesity (Silver Spring). 2014;22(12):2494-500.

63. Lien N, Bjelland M, Bergh IH, Grydeland M, Anderssen SA, Ommundsen Y, et al. Design of a 20-month comprehensive, multicomponent school-based randomised trial to promote healthy weight development among 11-13 year olds: The HEalth In Adolescents study. Scandinavian journal of public health. 2010;38(5 Suppl):38-51.

64. Llargues E, Franco R, Recasens A, Nadal A, Vila M, Perez MJ, et al. Assessment of a school-based intervention in eating habits and physical activity in school children: the AVall study. J Epidemiol Community Health. 2011;65(10):896-901.

65. Lowe MR, Feig EH, Winter SR, Stice E. Short-term variability in body weight predicts long-term weight gain. Am J Clin Nutr. 2015;102(5):995-9.

66. Lubans DR, Morgan PJ, Callister R. Potential moderators and mediators of intervention effects in an obesity prevention program for adolescent boys from disadvantaged schools. J Sci Med Sport. 2012;15(6):519-25.

67. Lubans DR, Morgan PJ, Dewar D, Collins CE, Plotnikoff RC, Okely AD, et al. The Nutrition and Enjoyable Activity for Teen Girls (NEAT girls) randomized controlled trial for adolescent girls from disadvantaged secondary schools: rationale, study protocol, and baseline results. BMC Public Health. 2010;10:652.

68. Lubans DR, Smith JJ, Plotnikoff RC, Dally KA, Okely AD, Salmon J, et al. Assessing the sustained impact of a school-based obesity prevention program for adolescent boys: the ATLAS cluster randomized controlled trial. The international journal of behavioral nutrition and physical activity. 2016;13:92.

69. MacKelvie KJ, McKay HA, Petit MA, Moran O, Khan KM. Bone mineral response to a 7-month randomized controlled, school-based jumping intervention in 121 prepubertal boys: associations with ethnicity and body mass index. J Bone Miner Res. 2002;17(5):834-44.

70. Madsen K, Thompson H, Adkins A, Crawford Y. School-community partnerships: a cluster-randomized trial of an after-school soccer program. JAMA pediatrics. 2013;167(4):321-6.

71. Magnusson KT, Hrafnkelsson H, Sigurgeirsson I, Johannsson E, Sveinsson T. Limited effects of a 2-year school-based physical activity intervention on body composition and cardiorespiratory fitness in 7-year-old children. Health Educ Res. 2012;27(3):484-94.

72. Martinez-Vizcaino V, Sanchez-Lopez M, Notario-Pacheco B, Salcedo-Aguilar F, Solera-Martinez M, Franquelo-Morales P, et al. Gender differences on effectiveness of a school-based physical activity intervention for reducing cardiometabolic risk: a cluster randomized trial. The international journal of behavioral nutrition and physical activity. 2014;11:154.

73. Mauriello LM, Ciavatta MM, Paiva AL, Sherman KJ, Castle PH, Johnson JL, et al. Results of a multi-media multiple behavior obesity prevention program for adolescents. Prev Med. 2010;51(6):451-6.

74. Mendoza JA, Baranowski T, Jaramillo S, Fesinmeyer MD, Haaland W, Thompson D, et al. Fit 5 Kids TV Reduction Program for Latino Preschoolers: A Cluster Randomized Controlled Trial. Am J Prev Med. 2016;50(5):584-92.

75. Mendoza JA, Watson K, Baranowski T, Nicklas TA, Uscanga DK, Hanfling MJ. The walking school bus and children's physical activity: a pilot cluster randomized controlled trial. Pediatrics. 2011;128(3):e537-44.

76. Meng L, Xu H, Liu A, van Raaij J, Bemelmans W, Hu X, et al. The costs and cost-effectiveness of a school-based comprehensive intervention study on childhood obesity in China. PLoS One. 2013;8(10):e77971.

77. Muckelbauer R, Libuda L, Clausen K, Kersting M. Long-term process evaluation of a school-based programme for overweight prevention. Child Care Health Dev. 2009;35(6):851-7.

78. Muzaffar H, Castelli DM, Scherer J, Chapman-Novakofski K. The impact of web-based HOT (Healthy Outcomes for Teens) Project on risk for type 2 diabetes: a randomized controlled trial. Diabetes technology & therapeutics. 2014;16(12):846-52.

79. Omwami EM, Neumann C, Bwibo NO. Effects of a school feeding intervention on school attendance rates among elementary schoolchildren in rural Kenya. Nutrition. 2011;27(2):188-93.

80. Patel AI, Grummon AH, Hampton KE, Oliva A, McCulloch CE, Brindis CD. A Trial of the Efficacy and Cost of Water Delivery Systems in San Francisco Bay Area Middle Schools, 2013. Preventing chronic disease. 2016;13:E88.

81. Perikkou A, Gavrieli A, Kougioufa MM, Tzirkali M, Yannakoulia M. A novel approach for increasing fruit consumption in children. J Acad Nutr Diet. 2013;113(9):1188-93.

82. Pinket AS, Van Lippevelde W, De Bourdeaudhuij I, Deforche B, Cardon G, Androutsos O, et al. Effect and Process Evaluation of a Cluster Randomized Control Trial on Water Intake and Beverage Consumption in Preschoolers from Six European Countries: The ToyBox-Study. PLoS One. 2016;11(4):e0152928.

83. Plachta-Danielzik S, Landsberg B, Lange D, Seiberl J, Muller MJ. Eight-year follow-up of school-based intervention on childhood overweight--the Kiel Obesity Prevention Study. Obes Facts. 2011;4(1):35-43.

84. Plachta-Danielzik S, Pust S, Asbeck I, Czerwinski-Mast M, Langnase K, Fischer C, et al. Four-year follow-up of school-based intervention on overweight children: the KOPS study. Obesity (Silver Spring). 2007;15(12):3159-69.

85. Powell CA, Walker SP, Chang SM, Grantham-McGregor SM. Nutrition and education: a randomized trial of the effects of breakfast in rural primary school children. Am J Clin Nutr. 1998;68(4):873-9.

86. Prina S, Royer H. The importance of parental knowledge: evidence from weight report cards in Mexico. J Health Econ. 2014;37:232-47.

87. Quinto Romani A. Estimating the peer effect on youth overweight and inactivity using an intervention study. J Sch Health. 2014;84(10):617-24.

88. Reed KE, Warburton DE, Macdonald HM, Naylor PJ, McKay HA. Action Schools! BC: a school-based physical activity intervention designed to decrease cardiovascular disease risk factors in children. Prev Med. 2008;46(6):525-31.

89. Reznik M, Wylie-Rosett J, Kim M, Ozuah PO. A classroom-based physical activity intervention for urban kindergarten and first-grade students: a feasibility study. Childhood obesity (Print). 2015;11(3):314-24.

90. Ridgers ND, Carter LM, Stratton G, McKenzie TL. Examining children's physical activity and play behaviors during school playtime over time. Health Educ Res. 2011;26(4):586-95.

91. Robbins LB, Ling J, Toruner EK, Bourne KA, Pfeiffer KA. Examining reach, dose, and fidelity of the "Girls on the Move" after-school physical activity club: a process evaluation. BMC Public Health. 2016;16:671.

92. Robinson TN. Can a school - based intervention to reduce television use decrease adiposity in children in grades 3 and 4 ? West J Med. 2000;173(1):40.

93. Rosario R, Oliveira B, Araujo A, Lopes O, Padrao P, Moreira A, et al. The impact of an intervention taught by trained teachers on childhood overweight. International journal of environmental research and public health. 2012;9(4):1355-67.

94. Rosenberg DE, Sallis JF, Conway TL, Cain KL, McKenzie TL. Active transportation to school over 2 years in relation to weight status and physical activity. Obesity (Silver Spring). 2006;14(10):1771-6.

95. Roth K, Kriemler S, Lehmacher W, Ruf KC, Graf C, Hebestreit H. Effects of a Physical Activity Intervention in Preschool Children. Med Sci Sports Exerc. 2015;47(12):2542-51.

96. Sacchetti R, Ceciliani A, Garulli A, Dallolio L, Beltrami P, Leoni E. Effects of a 2-year school-based intervention of enhanced physical education in the primary school. J Sch Health. 2013;83(9):639-46.

97. Safdie M, Jennings-Aburto N, Levesque L, Janssen I, Campirano-Nunez F, Lopez-Olmedo N, et al. Impact of a school-based intervention program on obesity risk factors in Mexican children. Salud Publica Mex. 2013;55 Suppl 3:374-87.

98. Saksvig BI, Webber LS, Elder JP, Ward D, Evenson KR, Dowda M, et al. A cross-sectional and longitudinal study of travel by walking before and after school among eighth-grade girls. J Adolesc Health. 2012;51(6):608-14.

99. Santos RG, Durksen A, Rabbanni R, Chanoine JP, Lamboo Miln A, Mayer T, et al. Effectiveness of peer-based healthy living lesson plans on anthropometric measures and physical activity in elementary school students: a cluster randomized trial. JAMA pediatrics. 2014;168(4):330-7.

100. Sarma KV, Udaykumar P, Balakrishna N, Vijayaraghavan K, Sivakumar B. Effect of micronutrient supplementation on health and nutritional status of schoolchildren: growth and morbidity. Nutrition. 2006;22(1 Suppl):S8-14.

101. Sazawal S, Habib A, Dhingra U, Dutta A, Dhingra P, Sarkar A, et al. Impact of micronutrient fortification of yoghurt on micronutrient status markers and growth - a randomized double blind controlled trial among school children in Bangladesh. BMC Public Health. 2013;13:514.

102. Shamah Levy T, Morales Ruan C, Amaya Castellanos C, Salazar Coronel A, Jimenez Aguilar A, Mendez Gomez Humaran I. Effectiveness of a diet and physical activity promotion strategy on the prevention of obesity in Mexican school children. BMC Public Health. 2012;12:152.

103. Sharma SV, Shegog R, Chow J, Finley C, Pomeroy M, Smith C, et al. Effects of the Quest to Lava Mountain Computer Game on Dietary and Physical Activity Behaviors of Elementary School Children: A Pilot Group-Randomized Controlled Trial. J Acad Nutr Diet. 2015;115(8):1260-71.

104. Simeon DT, Grantham-McGregor SM, Callender JE, Wong MS. Treatment of Trichuris trichiura infections improves growth, spelling scores and school attendance in some children. J Nutr. 1995;125(7):1875-83.

105. Sutherland R, Reeves P, Campbell E, Lubans DR, Morgan PJ, Nathan N, et al. Cost effectiveness of a multi-component school-based physical activity intervention targeting adolescents: the 'Physical Activity 4 Everyone' cluster randomized trial. The international journal of behavioral nutrition and physical activity. 2016;13:94.

106. Telford RD, Cunningham RB, Fitzgerald R, Olive LS, Prosser L, Jiang X, et al. Physical education, obesity, and academic achievement: a 2-year longitudinal investigation of Australian elementary school children. Am J Public Health. 2012;102(2):368-74.

107. Toulabi T, Khosh Niyat Nikoo M, Amini F, Nazari H, Mardani M. The influence of a behavior modification interventional program on body mass index in obese adolescents. J Formos Med Assoc. 2012;111(3):153-9.

108. van de Gaar VM, Jansen W, van Grieken A, Borsboom G, Kremers S, Raat H. Effects of an intervention aimed at reducing the intake of sugar-sweetened beverages in primary school children: a controlled trial. The international journal of behavioral nutrition and physical activity. 2014;11:98.

109. Van Lippevelde W, Bere E, Verloigne M, van Stralen MM, De Bourdeaudhuij I, Lien N, et al. The role of family-related factors in the effects of the UP4FUN school-based family-focused intervention targeting screen time in 10- to 12-year-old children: the ENERGY project. BMC Public Health. 2014;14:857.

110. Wang Y, Tussing L, Odoms-Young A, Braunschweig C, Flay B, Hedeker D, et al. Obesity prevention in low socioeconomic status urban African-american adolescents: study design and preliminary findings of the HEALTH-KIDS Study. Eur J Clin Nutr. 2006;60(1):92-103.

111. Watanabe J, Watanabe M, Yamaoka K, Adachi M, Nemoto A, Tango T. Effect of School-Based Home-Collaborative Lifestyle Education on Reducing Subjective Psychosomatic Symptoms in Adolescents: A Cluster Randomised Controlled Trial. PLoS One. 2016;11(10):e0165285.

112. Wendel ML, Benden ME, Zhao H, Jeffrey C. Stand-Biased Versus Seated Classrooms and Childhood Obesity: A Randomized Experiment in Texas. Am J Public Health. 2016;106(10):1849-54.

113. Whitt-Glover MC, Ham SA, Yancey AK. Instant Recess(R): a practical tool for increasing physical activity during the school day. Progress in community health partnerships : research, education, and action. 2011;5(3):289-97.

114. Williamson DA, Champagne CM, Harsha DW, Han H, Martin CK, Newton RL, Jr., et al. Effect of an environmental school-based obesity prevention program on changes in body fat and body weight: a randomized trial. Obesity (Silver Spring). 2012;20(8):1653-61.

115. Williamson DA, Copeland AL, Anton SD, Champagne C, Han H, Lewis L, et al. Wise Mind project: a school-based environmental approach for preventing weight gain in children. Obesity (Silver Spring). 2007;15(4):906-17.

116. Wilson DK, Lawman HG, Segal M, Chappell S. Neighborhood and parental supports for physical activity in minority adolescents. Am J Prev Med. 2011;41(4):399-406.

117. Xu F, Ware RS, Leslie E, Tse LA, Wang Z, Li J, et al. Effectiveness of a Randomized Controlled Lifestyle Intervention to Prevent Obesity among Chinese Primary School Students: CLICK-Obesity Study. PLoS One. 2015;10(10):e0141421.

118. Xu F, Ware RS, Tse LA, Wang Z, Hong X, Song A, et al. A school-based comprehensive lifestyle intervention among chinese kids against obesity (CLICK-Obesity): rationale, design and methodology of a randomized controlled trial in Nanjing city, China. BMC Public Health. 2012;12:316.

119. Yin Z, Gutin B, Johnson MH, Hanes J, Jr., Moore JB, Cavnar M, et al. An environmental approach to obesity prevention in children: Medical College of Georgia FitKid Project year 1 results. Obes Res. 2005;13(12):2153-61.

120. Yin Z, Moore JB, Johnson MH, Vernon MM, Gutin B. The impact of a 3-year after-school obesity prevention program in elementary school children. Childhood obesity (Print). 2012;8(1):60-70.

121. Zask A, Adams JK, Brooks LO, Hughes DF. Tooty Fruity Vegie: an obesity prevention intervention evaluation in Australian preschools. Health promotion journal of Australia : official journal of Australian Association of Health Promotion Professionals. 2012;23(1):10-5.
